# CoMnO_2_-Decorated Polyimide-Based Carbon Fiber Electrodes for Wire-Type Asymmetric Supercapacitor Applications

**DOI:** 10.3390/molecules25245863

**Published:** 2020-12-11

**Authors:** Young-Hun Cho, Jae-Gyoung Seong, Jae-Hyun Noh, Da-Young Kim, Yong-Sik Chung, Tae Hoon Ko, Byoung-Suhk Kim

**Affiliations:** 1 Department of Organic Materials & Fiber Engineering, Jeonbuk National University, 567 Baekje-daero, Deokjin-gu, Jeonju-si 54896, Jeollabuk-do, Korea; gamm7281@naver.com (Y.-H.C.); jaeging2@daum.net (J.-G.S.); nun20@naver.com (J.-H.N.); xxnn9@naver.com (Y.-S.C.); 2 Department of Polymer Nano Science & Technology, Jeonbuk National University, 567 Baekje-daero, Deokjin-gu, Jeonju-si 54896, Jeollabuk-do, Korea; psdcolor@jbnu.ac.kr

**Keywords:** carbon fiber, wire-type, CoMnO_2_, supercapacitor electrodes

## Abstract

In this work, we report the carbon fiber-based wire-type asymmetric supercapacitors (ASCs). The highly conductive carbon fibers were prepared by the carbonized and graphitized process using the polyimide (PI) as a carbon fiber precursor. To assemble the ASC device, the CoMnO_2_-coated and Fe_2_O_3_-coated carbon fibers were used as the cathode and the anode materials, respectively. Herein, the nanostructured CoMnO_2_ were directly deposited onto carbon fibers by a chemical oxidation route without high temperature treatment in presence of ammonium persulfate (APS) as an oxidizing agent. FE-SEM analysis confirmed that the CoMnO_2_-coated carbon fiber electrode exhibited the porous hierarchical interconnected nanosheet structures, depending on the added amount of APS, and Fe_2_O_3_-coated carbon fiber electrode showed a uniform distribution of porous Fe_2_O_3_ nanorods over the surface of carbon fibers. The electrochemical properties of the CoMnO_2_-coated carbon fiber with the concentration of 6 mmol APS presented the enhanced electrochemical activity, probably due to its porous morphologies and good conductivity. Further, to reduce the interfacial contact resistance as well as improve the adhesion between transition metal nanostructures and carbon fibers, the carbon fibers were pre-coated with the Ni layer as a seed layer using an electrochemical deposition method. The fabricated ASC device delivered a specific capacitance of 221 F g^−1^ at 0.7 A g^−1^ and good rate capability of 34.8% at 4.9 A g^−1^. Moreover, the wire-type device displayed the superior energy density of 60.2 Wh kg^−1^ at a power density of 490 W kg^−1^ and excellent capacitance retention of 95% up to 3000 charge/discharge cycles.

## 1. Introduction

In recent years, the energy storage devices, such as Li ion batteries (LIBs) and electrochemical capacitors/supercapacitors (SCs) are becoming more and more important as environmentally clean and sustainable energy sources [1,2,3]. In particular, SCs are the most promising energy storage device owing to their higher power densities, longer cycle life, fast charging/discharging capability, non-toxic nature, and low-cost maintenance compared to the LIBs. In general, the charge storage mechanism of supercapacitors is divided in two ways, either by forming electrical double layer charge accumulation (electrical double layer capacitors; EDLCs) or by using faradaic reactions (pseudocapacitor) at the interface between the electrode and electrolyte [2,4]. So far, various efforts have been made to meet increasing demand of high energy density and high-rate supercapacitors [2,5]. The development of the transition metal oxides-based pseudocapacitors with high specific capacitance values and excellent cycling stability, which can store and release the charges via the reversible redox reactions of metal oxide’s surface and inner sites [4,5], have practical value because transition metal oxides and hydroxides have higher theoretical specific capacitance and excellent energy density. In order to fulfill the growing energy density demands for next generation high-performance supercapacitors, the energy density (E = 0.5(CV^2^)) of supercapacitors can be improved by increasing the specific capacitance (C) and operation potential window (V). Accordingly, the electrode design of the pseudocapacitors with hierarchically nanostructured and battery-type bimetal oxides has been most important issues to improve the specific capacitance value and energy density. The extending of the potential window can be attained by the fabrication of asymmetric supercapacitors (ASCs), which effects an expanded potential window up to ~2V in aqueous electrolytes [6]. Nevertheless, the practical application of pseudocapacitors was still suffering from the limited energy and power densities, cycle lifetime and rate capability [7]. 

Recently, fiber-type flexible supercapacitors have been extensively studied due to the fast and steady progress in the portable/wearable electronics [8,9]. Till now, one-dimensional supercapacitors have been mostly studied based on fiber/yarn, cable, and wire as a current collector, due to the high flexibility, small size, lightweight, and easy to fabricate in wearable devices [10,11]. However, intensive researches are still required to further improve the performances of the fiber-based supercapacitors, such as energy and power densities, rate capability, and life span. Mostly, polymeric fibers [12,13] and carbon fibers [14,15,16] were used as a substrate to produce the fiber-type flexible supercapacitor devices, while nanocarbon materials (carbon nanotube, Ref. [12,17,18] graphene, Ref. [19,20] etc), transition metal oxides (NiCo_2_O_4_, MnO_2_, Fe_3_O_4_) [7,15,21], and conductive polymers (polyaniline [13], PEDOT [22,23], Polypyrrole [16]) were used as electroactive materials [15,24,25,26]. Most of studies have been devoted to solving the problems of lower capacitances and higher internal resistances between active materials and fiber-type electrodes, which can generally reduce the electrochemical performances for a practical application [27,28]. 

Here, we have used highly conductive carbon fibers as a wire-type electrode, which were prepared by the carbonized and graphitized process using the polyimide (PI) as a carbon fiber precursor. Briefly, the PI fibers were prepared by the wet spinning of polyamic acid and subsequent thermal imidization. The PI fibers were carbonized and finally graphitized at 2200 °C. Afterwards, the nanostructured CoMnO_2_ were directly deposited onto carbon fibers by a chemical oxidation route without high temperature treatments, which was carried out at moderately low temperature of 60 °C and did not need severe conditions, such as high temperature, toxic chemicals, complex manufacturing procedures, etc. Further, to reduce the interfacial contact resistance as well as improve the adhesion between transition metal nanostructures and carbon fibers, the carbon fibers were pre-coated with the Ni layer as a seed layer using an electrochemical deposition method. The assembled wire-type asymmetric supercapacitor device using CoMnO_2_-coated carbon fibers as the cathode and Fe_2_O_3_-coated carbon fibers as the anode materials delivered a specific capacitance of 221 F g^−1^ at 0.7 A g^−1^ and good rate capability of 34.8% at 4.9 A g^−1^. The fabricated wire-type flexible supercapacitor device displayed the superior energy density of 60.16 Wh kg^−1^ at a power density of 490 W kg^−1^ and excellent capacitance retention of 95% up to 3000 charge/discharge cycles. 

## 2. Results and Discussion

**Figure 1:**
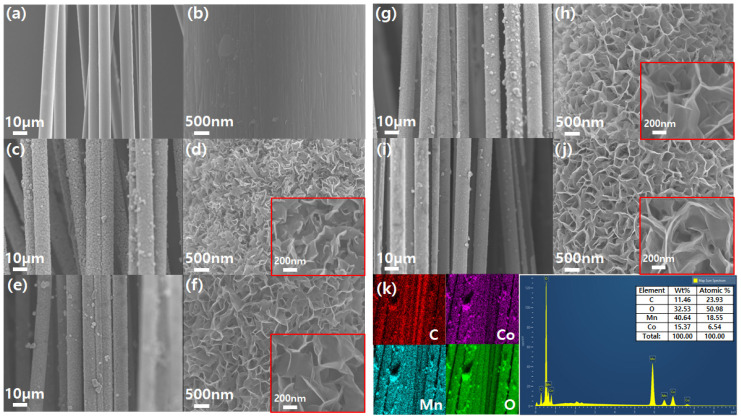
FE-SEM images of pure polyimide-based carbon fibers (PICFs) (**a**,**b**), CoMnO_2_@PICF-1 (**c**,**d**), CoMnO_2_@PICF-3 (**e**,**f**), CoMnO_2_@PICF-6 (**g**,**h**), and CoMnO_2_@PICF-9 (**i**,**j**), and inset shows the higher magnified images. The elemental mapping and EDX spectrum (**k**) of CoMnO_2_@PICF-6.

Figure 1 presents the FE-SEM images of pure PICF (Figure 1a,b), CoMnO_2_@PICF-1 (Figure 1c,d), CoMnO_2_@PICF-3 (Figure 1e,f), CoMnO_2_@PICF-6 (Figure 1g,h), and CoMnO_2_@PICF-9 (Figure 1i,j), respectively. It can be clearly seen that hierarchical CoMnO_2_ nanostructures were successfully formed on the surface of PICFs. While the bare PICFs showed smooth surface morphologies, CoMnO_2_@PICFs exhibited the porous hierarchical interconnected nanosheet structures, which depended on the added amounts of APS. Insets in Figure 1d–j show the higher magnified SEM images. We have also measured the elemental mapping and corresponding EDX spectrum using FE-SEM equipped with EDX measurement (Figure 1k). The results confirmed the presence of C, Co, Mn, and O elements, supporting the successful deposition of CoMnO_2_ onto PICFs.

**Figure 2:**
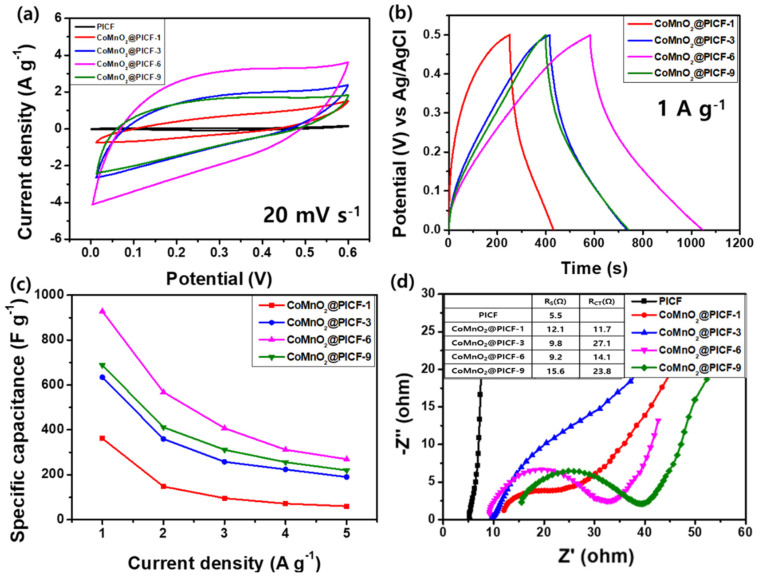
Cyclic voltammetry (CV) curves (**a**), galvanostatic charge–discharge (GCD) curves (**b**), relationship between capacitance and current density (**c**), and electrochemical impedance spectroscopy (EIS) curves (**d**) of pure PICFs, CoMnO_2_@PICF-1, CoMnO_2_@PICF-3, CoMnO_2_@PICF-6, and CoMnO_2_@PICF-9.

Figure 2a presents CV curves of pure PICFs, CoMnO_2_@PICF-1, CoMnO_2_@PICF-3, CoMnO_2_@PICF-6, and CoMnO_2_@PICF-9 at the scan rate of 20 mV s^−1^ within the potential window 0.0 to + 0.6 V vs. (Ag/AgCl). The shape of CV curves for CoMnO_2_@PICFs was nearly rectangular. No redox peaks were clearly detected, attributed to the fast, reversible successive surface redox reactions [29]. Moreover, it clearly showed that the integrated area of CoMnO_2_@PICF-6 was larger than those of CoMnO_2_@PICF-1, CoMnO_2_@PICF-3, and CoMnO_2_@PICF-9, suggesting feasible enhancement in the electrochemical activity, probably due to its porous morphology and good conductivity. On the other hand, the contribution of pure PICFs to the capacitance was almost negligible, as seen in Figure 2a, suggesting that the added CoMnO_2_ significantly enhanced the capacitance. Further, as increasing the scan rate the CV curves of CoMnO_2_@PICFs well maintained symmetrical shape (Figure S1), demonstrating the reversible electrochemical redox reaction and excellent rate capability of the electrode material [30], which is one of important parameters in the pseudocapacitive electrodes.

Capacitive performance of the CoMnO_2_@PICFs electrodes was further studied by the GCD measurements at the current density of 1 A g^−1^ within the potential range from 0 to + 0.5V (Figure 2b). The fabricated CoMnO_2_@PICF-1, CoMnO_2_@PICF-3, CoMnO_2_@PICF-6, and CoMnO_2_@PICF-9 electrodes delivered the specific capacitances of 362, 634, 928, and 688 F g^−1^ at the current density of 1 A g^−1^, respectively. Figure 2c shows the relationship of specific capacitance vs. current density of the CoMnO_2_@PICF electrodes. The specific capacitance (~928 F g^−1^@1 A g^−1^) of the CoMnO_2_@PICF-6 electrode was about 2.6, 1.5, and 1.3 times higher than those of the CoMnO_2_@PICF-1 (~362 F g^−1^@1 A g^−1^), CoMnO_2_@PICF-3 (~643 F g^−1^@1 A g^−1^), and CoMnO_2_@PICF-9 (~688 F g^−1^@1 A g^−1^) electrodes at the current density of 1.0 A g^−1^, respectively. Moreover, the capacitance of the CoMnO_2_@PICF-6 electrode decreased by 29 % at higher current density of 5 A g^−1^, proving the excellent high-rate capability. The EIS was exploited to investigate the ion diffusion and electron transfer of the CoMnO_2_@PICF electrodes. Figure 2d presents the Nyquist plots for the pure PICF, CoMnO_2_@PICF-1, CoMnO_2_@PICF-3, CoMnO_2_@PICF-6, and CoMnO_2_@PICF-9 electrodes in the frequency range of 0.1 to 100 kHz with an amplitude of 10 mV. The EIS spectrum was composed of a small semicircle in a high-frequency range and a linear curve in the low-frequency range. The internal resistance (R_S_) is the sum of the ionic resistance of the electrolyte. The intrinsic resistance of the active material and the contact resistance at the active material/current collector interface can be derived from the intercept of the plots on the real axis. The semicircle of Nyquist plot corresponds to the Faradic reaction and its diameter represents the interfacial charge transfer resistance (R_CT_) [31]. The fabricated CoMnO_2_@PICFs electrodes showed a semicircle at higher frequency region. The calculated R_CT_ values were 11.7 Ω, 27.1 Ω, 14.1 Ω and 23.8 Ω for the CoMnO_2_@PICF-1, CoMnO_2_@PICF-3, CoMnO_2_@PICF-6, and CoMnO_2_@PICF-9 electrodes, respectively. The values were tabulated in inset of figure. As a result, it was found that the CoMnO_2_@PICF-6 showed best electrochemical activity. Although the value of R_CT_ (14.1 Ω) of the CoMnO_2_@PICF-6 electrode was a rather large, we could see the lower R_S_ value (9.2 Ω) of the CoMnO_2_@PICF-6 electrode at the intersection of the real axis, indicating a low internal resistance.

**Figure 3:**
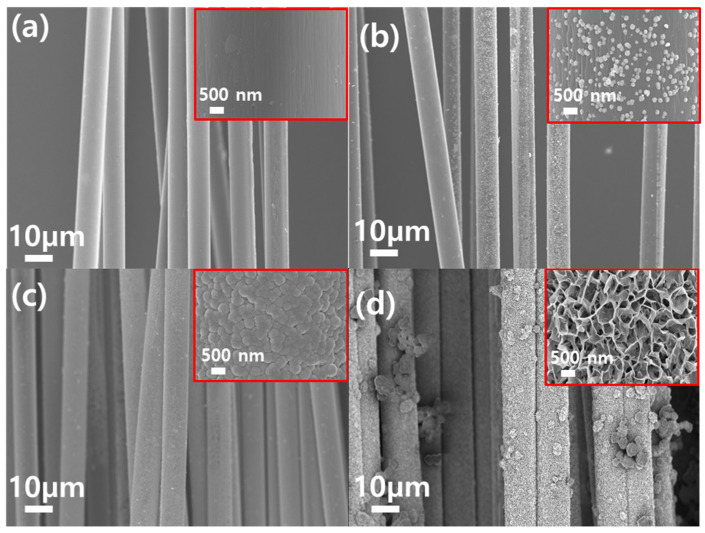
FE-SEM images of pure PICF (**a**), N10@PICF (**b**), N20@PICF (**c**), and CM/N20@PICF-6 (**d**), inset shows the higher magnified images.

**Figure 4:**
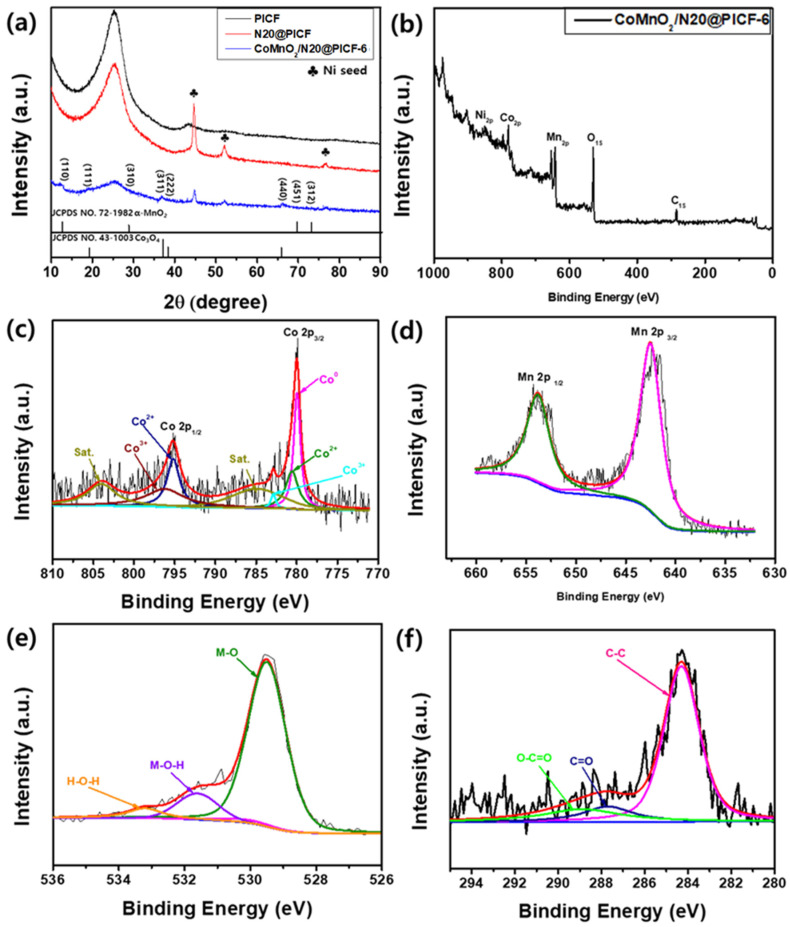
XRD patterns (**a**) of pure PICF, N20@PICF and CoMnO_2_/N20@PICF-6. The XPS survey spectrum (**b**) of CoMnO_2_/N20@PICF-6 and XPS spectra of Co 2p (**c**), Mn 2p (**d**), O 1s (**e**), C 1s (**f**) for CoMnO_2_/N20@PICF-6 in high resolution.

In order to further improve the interfacial properties of the fabricated CoMnO_2_@PICFs electrode, the additional Ni as a seed layer was deposited on the PICF before CoMnO_2_ deposition. Figure 3 shows the FE-SEM images of pure PICF (Figure 3a), N10@PICF (Figure 3b), N20@PICF (Figure 3c), and CoMnO_2_/N20@PICF-6 (Figure 3d), respectively. This result indicated that N20@PICF showed evenly Ni-coated and rather smooth surface morphology (Figure 3c), while N10@PICF clearly showed poor coverage of Ni on PICF (Figure 3b). Furthermore, CoMnO_2_/N20@PICF-6 showed the similar surface morphology to the CoMnO_2_@PICF-6 (Figure 3d). We have checked the crystal structure of pure PICF, N20@PICF, and CoMnO_2_/N20@PICF-6. As seen in Figure 4a, bare PICF showed a broad peak around 25.4^o^, corresponding to the graphitic carbon peak [32]. The Ni20@PICF showed the three obvious diffraction peaks appearing at 44.6^o^, 52.0^o^, and 76.7^o^, which were well indexed to the (111), (200), and (220) crystal planes of the face-centered cubic structure of nickel/nickel oxide (JCPDS No. 87-0712) [33] originated from the 3D-Ni metal skeleton [21] and coated Ni seed layer. The CoMnO_2_/N20@PICF-6 showed the diffraction peaks at 12.7°, 18.9°, 28.7°, 36.8°, 38.3°, 65.9°, 69.6°, and 73.1°, which were well indexed to the (110), (111), (310), (311), (222), (440), (451), and (312) crystal planes, respectively. Among them, the diffraction peaks at 12.2°, 28.7°, 69.6°, and 73.1^o^ were indexed to the characteristic (110), (310), (451), and (312) crystal planes of α-MnO_2_ [JCPDS No. 72-1982] [34]. The other peaks at 18.9^o^, 36.8^o^, 38.3°, and 65.9° were well indexed to the characteristic (111), (311), (222), and (440) crystal planes of Co_3_O_4_ (JCPDS No. 43-1003) [35]. The fitted lattice parameter of α-MnO_2_ and Co_3_O_4_ were a = 9.816Å and a = 8.076Å, which were perfectly matched with standard JCPDS No. 72-1982 and JCPDS No. 43-1003, respectively. These results demonstrated the successful coating of CoMnO_2_ with amorphous phase onto the PICF [36].

To confirm the chemical states of CoMnO_2_/N20@PICF-6, XPS studies were carried out (Figure 4b–f). The survey spectrum shows the presence of Ni, Co, Mn, O, and C without other impurity elements. The observed Ni peak is ascribed to the Ni seed layer on the PICF. The deconvoluted Co 2p spectrum suggested the existence of Co^(0)^, Co^2+^, and Co^3+^ at the 778.5–795.1 eV, 780.5 eV, and 779.5–797.3 eV, respectively. The deconvoluted Mn 2p spectrum displayed two peaks at 653.9 and 642.2 eV, which were ascribed to the presence of mixed Mn 2p^1/2^ and Mn 2p^3/2^ with a spin-energy separation of 11.7eV. The O 1s spectrum at 529.5 eV, 531.58 eV, and 533.28 eV can be attributed to metal oxygen bond (M-O, M-Co, and Mn), the hydrated trivalent oxide bond (M-O-H), and the residual water bond (H-O-H), respectively. In the C 1s spectrum of CoMnO_2_@PICFs, the sharp peak at around 286 eV can be spilt to three peaks at 284.4, 286.6, and 288.9 eV, which represent the sp^2^ graphitic carbon, C–O and O–C=O bonds. The Co and Mn contents of CoMnO_2_/N20@PICFs reached 15.87 and 14.57 atomic%, suggesting the successful formation of CoMnO_2_ on the fiber substrate.

**Figure 5:**
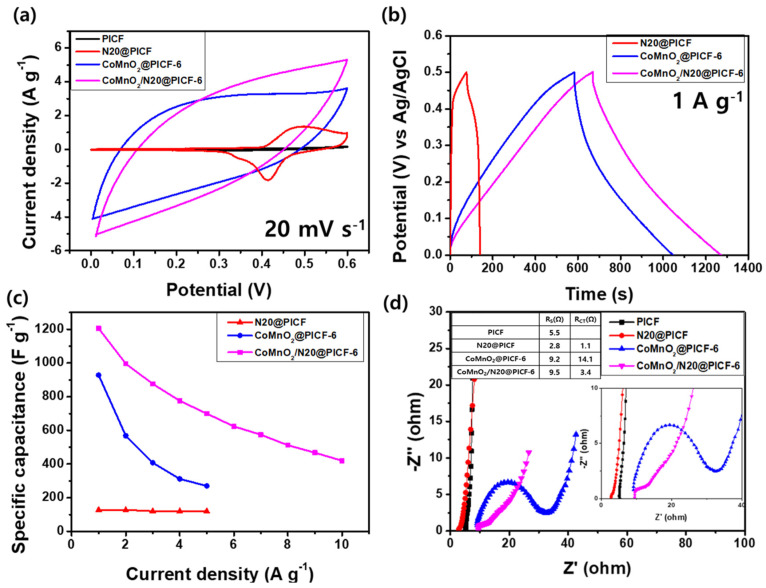
CV curves (**a**), GCD curves (**b**), specific capacitance vs. current density (**c**), Nyquist plot (**d**) of bare PICF, N20@PICF, CoMnO_2_@PICF-6 and CoMnO_2_/N20@PICF-6.

In order to investigate the effect of Ni seed layer on the electrochemical properties of CoMnO_2_@PICF-6, CV and EIS tests of bare PICF, CoMnO_2_@PICF-6, and CoMnO_2_/N20@PICF-6 were carried out in three-electrode configuration. As seen in Figure 5a, the CoMnO_2_/N20@PICF-6 electrode showed clearly large integrated area than those of other samples, suggesting an enhanced electrochemical storage ability. As expected, the CoMnO_2_/N20@PICF-6 displayed the longer discharge time (Figure 5b), as compared to the CoMnO_2_@PICF-6. The specific capacitances calculated from the discharge curves were plotted as a function of current density (Figure 5c). The CoMnO_2_/N20@PICF-6 electrode delivered excellent specific capacitances of 1206, 996, 876, 776, 700, 624, 574, 512, 468, and 420 F g^−1^ at the current densities of 1, 2, 3, 4, 5, 6, 7, 8, 9, and 10 A g^−1^, respectively, which were clearly higher than those of the CoMnO_2_@PICF-6 and even N20@PICF electrodes. The electrochemical performances of the CoMnO_2_-decorated PICF electrodes (CoMnO_2_@PICF-6, CoMnO_2_/N20@PICF-6) were further investigated by EIS. Compared to the CoMnO_2_@PICF-6 (14.1 Ω), the CoMnO_2_/N20@PICF-6 showed the lower R_CT_ value (3.4 Ω), suggesting that Ni seed layer provided a good interfacial contact between CoMnO_2_ and PICF, giving the lower interfacial resistance and a fast reversible redox reaction.

**Figure 6:**
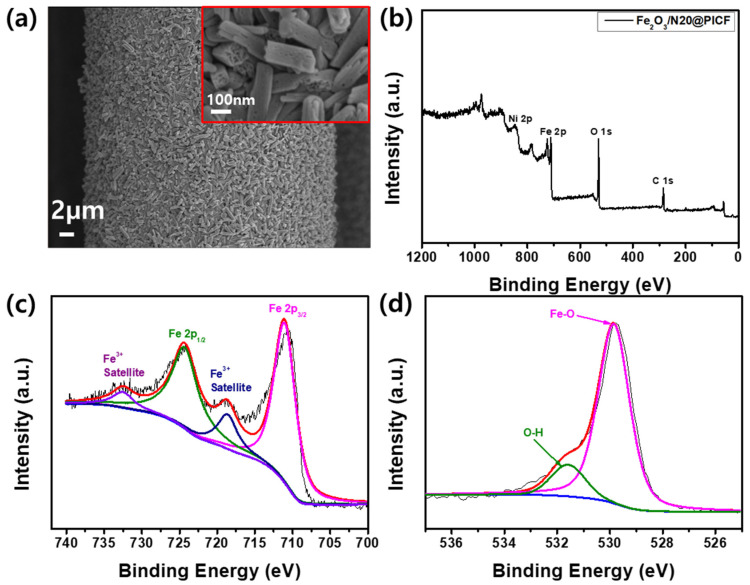
FE-SEM image (**a**) XPS survey spectrum (**b**) and XPS spectra of Fe 2p (**c**) O 1s (**d**) for Fe_2_O_3_/N20@PICF in high resolution.

To evaluate the real capacitance of the fabricated electrodes, an ASC full-cell was assembled with cathode and anode materials. To fabricate the full-cell, Fe_2_O_3_-decorated N20@PICF with Ni seed layer (Fe_2_O_3_/N20@PICF) was prepared by hydrothermal method and then used as anode material. FE-SEM image showed a uniform distribution of porous Fe_2_O_3_ nanorods over the surface of PICF, as can be seen in inset of Figure 6a. Figure 6b shows the survey XPS spectrum of Fe_2_O_3_/N20@PICF sample which contained Fe, O, Ni, and C elements. The Fe 2p spectrum (Figure 6c) exhibited two distinct peaks of Fe 2p^1/2^ and Fe 2p^3/2^ at the binding energies of 724.6 and 710.9 eV, respectively. The two satellite peaks were also observed, indicating the existence of Fe^3+^ in Fe_2_O_3_ [37]. Figure 6d presented the XPS spectrum of O 1s level of the sample. The peak can be deconvoluted into two peaks at 529.8 and 531.6 eV. The peak at 529.8 eV was due to the lattice oxygen of the Fe_2_O_3_, and the other peak at the 531.6 eV represented the presence of other components such as OH, H_2_O, and carbonate species adsorbed onto the surface [38]. The porous structures in electroactive materials also plays an important role in electrochemical energy storage devices. Figure S2 shows the N_2_ adsorption/desorption curves of CoMnO_2_/N20@PICF-6 electrode. It exhibited a type IV isotherm with an H3 hysteresis loop at high relative pressure, indicating the presence of mesoporous structure. The BET surface area and total pore volume were measured to be 27.54 m^2^·g^−1^ and 0.048 cm^3^·g^−1^. The result showed that the major volume of the pores was the mesopore, ranging from of 1.9 to 4.2 nm. Therefore, the large specific surface area and the mesoporous architecture of the CoMnO_2_/N20@PICF-6 electrode can be expected to provide enormous electroactive sites at the electrode/electrolyte interface and shorten the ion diffusion length for an excellent electrochemical performance [39].

**Figure 7:**
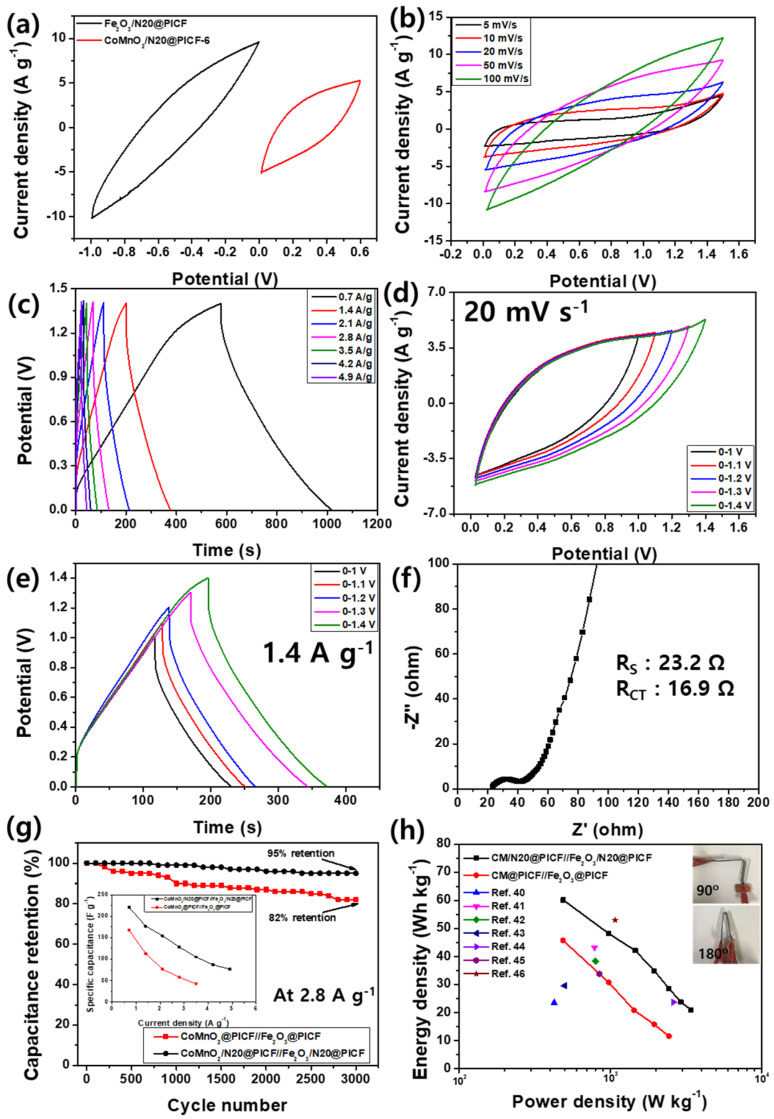
CV curves (**a**) of the cathode and anode materials at 20 mV s^−1^, CV curves (**b**) of the asymmetric supercapacitors (ASC) device at various scan rates, GCD curves (**c**) of the ASC device at different currents density, CV curves (**d**) at different potential windows at 20 mV s^−1^, GCD curves (**e**) at different potential windows at 1.4 A g^−1^, Nyquist plot (**f**) of the ASC device, cycle performance (**g**) of the ASC device for 3000 cycles at 2.8 A g^−1^ (inset shows the relationship between specific capacitance and current density of the ASC device), (**h**) Ragone plot (inset shows the digital images of the ASC device upon bending).

To test the feasibility of the CoMnO_2_/N20@PICF-6 and Fe_2_O_3_/N20@PICF electrodes for a real application, we have assembled the ASC device by sandwiching the CoMnO_2_/N20@PICF-6 as a cathode and Fe_2_O_3_/N20@PICF as an anode material with PVA-KOH as a polymer electrolyte. The optimal mass ratio (m^+^/m^−^) of the cathode and anode materials was calculated by using Equation (2), and it turned out to be 1:0.7. The CV profiles of the CoMnO_2_/N20@PICF6 and Fe_2_O_3_/N20@PICF electrodes were shown in Figure 7a. The potential windows of the anode and cathode materials were −1.0 to 0 V and 0 to 0.6 V, respectively. Accordingly, the maximum operation potential for the wire-type ASC device was anticipated to reach 1.5 V. The typical CV curves of the ASC device in a potential window of 0.0–1.5 V at different scan rates were presented in Figure 7b. It displayed a rectangular-like shape, indicating that the device showed a fast charge/discharge behavior and high rate ability. The triangular-shaped GCD curves in Figure 7c revealed the satisfactory electrochemical reversibility and capacitive characteristics of the ASC device. The calculated specific capacitance was 221 F g^−1^ at the current density of 0.7 A g^−1^. Moreover, the capacitance value started to decrease as the current density further increased. The ASC device showed stable electrochemical performances at different voltage windows, as seen in Figure 7d. The CV profiles remained rectangular-like shape up to 1.4 V. The charge–discharge profiles at different voltage ranges also exhibited triangular-shaped curves up to 1.4 V (Figure 7e). With the extension of voltage windows, the calculated electrical performance of the device slightly increased from 159.6 F g^−1^ to 177 F g^−1^. The Nyquist plot (Figure 7f) showed the estimated R_S_ and R_CT_ values of 23.2 Ω and 16.9 Ω, respectively, which derived from the low resistance of the conductive carbon fiber substrate and the compact cell assembly. At low frequencies, the ASC device showed vertical behavior, which indicates that samples possess capacitor characteristics. Thus, the ASC device had good capacitive properties. In addition, the CoMnO_2_/N20@PICF-6//Fe_2_O_3_/N20@PICF device exhibited higher capacitance retention rate of 95% after 3000 charge/discharge cycles at 2.8 A g^−1^ than that (retention rate of 82%) of the CoMnO_2_@PICF-6//Fe_2_O_3_@PICF device without Ni seed layer, ascribed to the reduced interfacial contact resistance by an introduced Ni layer (Figure 7g). Furthermore, the rate capability was enhanced by the electrodeposition of Ni seed layer (inset in Figure 7g). The assembled CoMnO_2_/N20@PICF-6//Fe_2_O_3_/N20@PICF device delivered a maximum energy density of 60.2 Wh kg^−1^ at power density of 490 W kg^−1^, as shown by Ragone plot (Figure 7h). Compared to other devices, these values were higher than other energy storage devices, such as NiCoMn-TH/AEG//CFP-S (23.5 Wh kg^−1^ at 427 W kg^−1^) [40], NiCoMn-OH//AC (43.2 Wh kg^−1^ at 790 W kg^−1^) [41], CoMn-HW/RGO10//AC (38.3 Wh kg^−1^ at 8000 W kg^−1^) [42], CoMn LDH/PPy//MLG (29.6 Wh kg^−1^ at 500 W kg^−1^) [43], NCM//AC (23.7 Wh kg^−1^ at 2625 W kg^−1^) [44], Ni-Mn LDH/rGO//AC (33.8 Wh kg^−1^ at 850 W kg^−1^) [45], and Co/Mn-ZIF//AC (52.5 Wh kg^−1^ at 1080 W kg^−1^) [46], as summarized in Table S1. As a result, the fabricated wire-type ASC device exhibited the excellent electrochemical performance with good electrical conductivity.

## 3. Materials and Methods

### 3.1. Materials

Poly(amic acid) (PAA, composed of pyromellitic dianhydride (PDMA)/4,4′-diaminodiphenyl ether (4,4′-ODA), 230,000 cPs of viscosity, PI Advanced Materials Co., Ltd., Anyang, Korea)-based carbon fibers (PICFs) [47] were kindly provided by Dissol Inc., Republic of Korea. Cobalt(II) acetate tetrahydrate (Co(CH_3_COO)_2_·4H_2_O), manganese(II) acetate tetrahydrate (Mn(CH_3_COO)_2_·4H_2_O), iron(III) chloride hexahydrate(FeCl_3_·6H_2_O), nickel(II) chloride hexahydrate (NiCl_2_·6H_2_O), ammonium persulfate (APS, (NH_4_)_2_S_2_O_8_)), and ammonium chloride (NH_4_Cl) were purchased from Sigma-Aldrich (St. Louis, MO, USA). All the reagents were an analytical grade and used without further purification. All aqueous solutions used in these experiments were prepared with deionized (DI) water (18.2 MΩ⋅cm, Elga DI water system, Woodridge, VA, USA).

### 3.2. Fabrication of Transition Metal Oxides-Coated PICF Electrodes

At first, 4.5 mmol Co(CH_3_COO)_2_·4H_2_O and 4.5 mmol Mn(CH_3_COO)_2_·4H_2_O were dissolved in 30 mL of DI water and stirred for 1 h to make a clear bright red solution. Then the PICF was immersed in the above solution and sonicated slightly to remove the microbubbles of the solution. Afterwards, APS solution with various concentrations of 1, 3, 6, and 9 mmol was further added and reacted at 60 °C for 12 h (via a chemical oxidation reaction) and labelled as CoMnO_2_@PICF-1, CoMnO_2_@PICF-3, CoMnO_2_@PICF-6, and CoMnO_2_@PICF-9, respectively. The obtained CoMnO_2_-decorated PICFs (CoMnO_2_@PICFs) as cathode material was washed with DI water gently, and dried at 60 °C. The mass loading of CoMnO_2_ metal oxide deposited onto PICF was about 2 mg. For the preparation of anode material, 6 mmol FeCl_3_·6H_2_O was dissolved in 60 mL of DI water and stirred for 1 h at room temperature to make a homogeneous solution. Then, PICF and the above solution were transferred into 80 mL Teflon-lined stainless steel autoclave, and hydrothermal treatment was carried out at 140 °C for 12 h in an oven. The resultant product was carefully rinsed with DI water and dried at 60 °C for 12 h. Finally, the obtained sample was further calcinated at 350 °C for 2 h at a heating rate of 2 °C min^−1^ to achieve the Fe_2_O_3_-decorated PICF (Fe_2_O_3_@PICF). The mass loading of Fe_2_O_3_ metal oxides deposited onto PICF was about 1 mg.

### 3.3. Electrodeposition of Ni Seed Layer on PICF

**Scheme 1:**
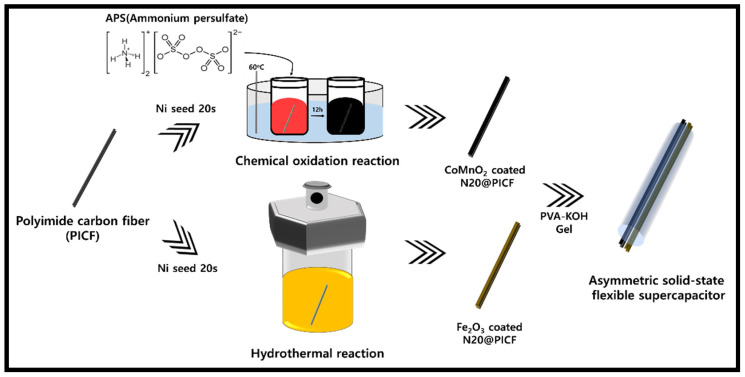
Schematic illustration for the preparation of a wire-type asymmetric supercapacitor by using CoMnO_2_- and Fe_2_O_3_-coated carbon fibers as cathode and anode materials.

For the deposition of Ni seed layer, 2M NH_4_Cl and 0.1M NiCl_2_·6H_2_O as a supporting electrolyte were dissolved in 100 mL of DI water, and used as the solution for electrodeposition. The PICF and Pt wire were used as working and reference electrodes, respectively. The Ni seed layer deposition was carried out with a constant current density of 0.25 A cm^−2^ (by adjusting 7 V and 0.5 A) at a deposition time of 20 s (denoted as Ni20@PICF) using a DC power supply. After deposition, the samples were washed with DI water and dried at 60 °C for 12 h. The schematic illustration for the preparation of CoMnO_2_@PICFs was represented in Scheme 1.

### 3.4. Characterization

The surface morphologies were examined by field emission scanning electron microscopy (FE-SEM, JEOL JSM-5900) along with energy dispersive X-ray (EDX) system. The X-ray diffraction studies were performed using Rigaku diffractometer, CuK_α_ radiation operating at 40 keV/40 mA at a scanning rate of 15° per min in the 2θ range from 10° to 90°. The chemical state of the elements linked with the surface chemical composition of the sample was analyzed by X-ray photoelectron spectroscopy (XPS, ESCALAB250, Al K_α_ radiation). The specific surface area (SSA) and pore size of the samples were computed using the Brunauer–Emmett–Teller (BET) equation.

### 3.5. Electrochemical Measurements

The electrochemical performance was investigated by cyclic voltammetry (CV), electrochemical impedance spectroscopy (EIS), and galvanostatic charge–discharge (GCD). The CV and EIS measurements were carried out in a three-electrode system at room temperature using an electrochemical workstation (Princeton Applied Research, Versatat 4). Here, Pt wire was used as the counter electrode, Ag/AgCl as the reference electrode and the prepared samples as the working electrode, respectively. The CV curves were recorded within the potential window from 0 to 0.6 V (vs. Ag/AgCl) in 1M KOH electrolyte at various scan rates (5, 10, 20, 50, and 100 mV s^−1^). The GCD tests were carried out within the potential range of 0 to 0.5 V in 1M KOH. The specific capacitance of the fabricated electrodes was calculated from the discharge curves using the following Equation (1).

C = IΔt/mΔV(1)

where C is the specific capacitance (F g^−1^), I is the discharge current (mA), m is the mass (mg) of the electroactive material, ∆t and ∆V are the discharge time (s) and potential window (V). The EIS measurement was carried out at open circuit potential in the frequency range of 0.1 Hz to 100 kHz. The ZView software was employed to fit the EIS data. The impedance data are presented in the form of Nyquist plot, representing the resistive and capacitive behavior of electrodes. The values of the charge transfer resistance (R_CT_) and internal resistance (R_S_) were determined using Zsimpwin software simulations.

For the practical applications, the asymmetric supercapacitor (ASC, CoMnO_2_/N20@PICF//Fe_2_O_3_/N20@PICF) device was constructed using CoMnO_2_/N20@PICF and Fe_2_O_3_/N20@PICF as cathode and anode materials, respectively. The PVA/KOH gel electrolyte was used for both electrolyte and separator in ASC device. To prepare the PVA/KOH gel electrolyte, 2 g PVA and 2g KOH were added to the 20 mL DI water and it was kept under stirring and then heated slowly to 90 °C until it became clear and transparent. After it was cooled down to room temperature, the CoMnO_2_/N20@PICF and Fe_2_O_3_/N20@PICF electrodes were coated with PVA/KOH gel electrolyte and carefully assembled together and then sealed in the plastic tube to fabricate the wire-type ASC device. To obtain optimum energy and power densities, the optimal mass ratio of both electrodes was determined based on the charge balance relationship (q^+^ and q^-^ are the charges acquired by the cathode and anode materials) provided by the following Equation (2).

m^+^/m^−^ = (C^−^ × V^−^)/(C^+^ × V^+^) (2)

where m^+^, m^−^, C^+^, C^−^, V^+^, and V^−^ signifies the mass (g), specific capacitance (F g^−1^) and working potential window (V) for the cathode and anode materials, respectively. The energy (E, Wh kg^−1^) and power densities (P, W kg^−1^) of as-assembled ASC device were computed by the following Equations (3) and (4).

E = C × ∆V^2^/2 × 3.6 (3)

P = E × 3600/∆t(4)

where C indicates the specific capacitance of ASC device (F g^−1^), ΔV is the working potential window (V) and Δt is the discharging time (s).

## 4. Conclusions

We have prepared the carbon fiber-based wire-type asymmetric supercapacitors (ASCs). The nanostructured and porous CoMnO_2_-coated and Fe_2_O_3_-coated carbon fibers were used as the cathode and the anode materials to produce the asymmetric CoMnO_2_/N20@PICF-6//Fe_2_O_3_/N20@PICF supercapacitor device. Such porous hierarchical interconnected nanosheet structures were confirmed by FE-SEM analysis. The electrochemical properties of the CoMnO_2_-coated carbon fiber electrode (CoMnO_2_@PICF-6) with the concentration of 6 mmol APS presented the enhanced electrochemical activity, due to its porous morphologies and good conductivity. Moreover, additional Ni seed layer provided a good interfacial contact between CoMnO_2_ and PICF, giving the lower interfacial resistance and a fast reversible redox reaction. The fabricated ASC device delivered a specific capacitance of 221 F g^−1^ at 0.7 A g^−1^ and good rate capability of 34.8% at 4.9 A g^−1^. The wire-type device displayed the superior energy density of 60.2 Wh kg^−1^ at a power density of 490 W kg^−1^ and excellent capacitance retention of 95% up to 3000 charge/discharge cycles.

## Supplementary Materials

The following are available online, Figure S1: CV curves of CoMnO_2_@PICF-1, CoMnO_2_@PICF-3, CoMnO_2_@PICF-6, and CoMnO_2_@PICF-9 electrode materials at various scan rates, Figure S2; N2 adsorption/desorption curve (Figure S2a) and the pore size distribution curve (Figure S2b) of CoMnO_2_/N20@PICF-6 electrode, Table S1: The specific capacitance, energy density, cycle stability, and electrolyte of the CoMnO_2_/N20@PICF-6//Fe_2_O_3_/N20@PICF device, compared to previously reported transition metal oxides based electrode materials.

## References

[1] Wang L., Menakath A., Han F., Wang Y., Zavalij P.Y., Gaskell K.J., Borodin O., Iuga D., Brown S.P., Wang C. (2019). Identifying the components of the solid-electrolyte interphase in Li-ion batteries. Nat. Chem..

[2] Zhai S., Karahan H.E., Wang C., Pei Z., Wei L., Chen Y. (2020). 1D supercapacitors for emerging electronics: Current status and future directions. Adv. Mater..

[3] Yaghtin A., Masoudpanah S.M., Hasheminiasari M., Salehi A., Safanama D., Ong C.K., Adams S., Reddy M.V. (2020). Effect of reducing agent on solution synthesis of Li_3_V_2_(PO_4_)_3_ cathode material for lithium ion batteries. Molecules.

[4] Zhou K., Zhou W., Yang L., Lu J., Cheng S., Mai W., Tang Z., Li L., Chen S. (2015). Ultrahigh-performance pseudocapacitor electrodes based on transition metal phosphide nanosheets array via phosphorization: A general and effective approach. Adv. Funct. Mater..

[5] Vijayan B.L., Zain N.K.M., Misnon I.I., Reddy M.V., Adams S., Yang C.C., Anilkumar G.M., Jose R. (2020). Void space control in porous carbon for high-density supercapacitive charge storage. Energy Fuels.

[6] Tang X., Jia R., Zhai T., Xia H. (2015). Hierarchical Fe_3_O_4_@Fe_2_O_3_ core-shell nanorod arrays as high-performance anodes for asymmetric supercapacitors. ACS. Appl. Mater. Int..

[7] Harilal M., Krishnan S.G., Vijayan B.L., Reddy M.V., Adams S., Barron A.R., Yusoff M.M., Jose R. (2017). Continuous nanobelts of nickel oxide-cobalt oxide hybrid with improved capacitive charge storage properties. Mat. Des..

[8] Chodankar N.R., Dubal D.P., Ji S.H., Kim D.H. (2019). Self-assembled nickel pyrophosphate-decorated amorphous bimetal hydroxides 2D-on-2D nanostructure for high-energy solid state asymmetric supercapacitor. Small.

[9] Patil A.M., Lokhande A.C., Chodankar N.R., Shinde P.A., Kim J.H., Lokhande C.D. (2017). Interior design engineering of CuS architecture alteration with rise in reaction bath temperature for high performance symmetric flexible solid state supercapacitor. J. Ind. Eng. Chem..

[10] Meng F., Li Q., Zheng L. (2017). Flexible fiber-shaped supercapacitors: Design, fabrication, and multi-functionalities. Energy Storage Mater..

[11] Hu M., Liu Y., Zhang M., Wei H., Gao Y. (2016). Wire-type MnO_2_/Multilayer graphene/Ni electrode for high-performance supercapacitor. J. Power Sources.

[12] Yang Z., Deng J., Chen X., Ren J., Peng H. (2013). A highly stretchable, fiber-shaped supercapacitor. Angew. Chem. Int. Ed..

[13] Nwanya A.C., Jafta C.J., Ejikeme P.M., Ugwuoke P.E., Reddy M.V., Osuji R.U., Ozoemena K.I., Ezema F.I. (2014). Electrochromic and electrochemical capacitive properties of tungsten oxide and its polyaniline nanocomposite films obtained by chemical bath deposition method. Electrochim. Acta.

[14] Liu N., Ma W., Tao J., Zhang X., Su J., Li L., Yang C., Gao Y., Golberg D., Bando Y. (2013). Cable-type supercapacitors of three-dimensional cotton thread based multi-grade nanostructures for wearable energy storage. Adv. Mater..

[15] Zhai T., Xie S., Yu M., Fang P., Liang C., Lu X., Tong Y. (2014). Oxygen vacancies enhancing capacitive properties of MnO_2_ nanorods for wearable asymmetric supercapacitors. Nano Energy.

[16] Ko T.H., Lei D., Balasubramaniam S., Seo M.K., Chung Y.S., Kim H.Y., Kim B.S. (2017). Polypyrrole-decorated hierarchical NiCo_2_O_4_ nanoneedles/carbon fiber papers for flexible high-performance supercapacitor applications. Electrochim. Acta.

[17] Jayaseelan S.S., Ko T.H., Radhakrishnan S., Yang C.M., Kim H.Y., Kim B.S. (2016). Novel MWCNT interconnected NiCo_2_O_4_ aerogels prepared by a supercritical Co_2_ drying method for ethanol electrooxidation in alkaline media. Int. J. Hydrogen Energy.

[18] Wang K., Meng Q., Zhang Y., Wei Z., Miao M. (2013). High-performance two-ply yarn supercapacitors based on carbon nanotubes and polyaniline nanowire arrays. Adv. Mater..

[19] Meng Y., Zhao Y., Hu C., Cheng H., Hu Y., Zhang Z., Shi G., Qu L. (2013). All-graphene core-sheath microfibers for all-solid-state, stretchable fibriform supercapacitors and wearable electronic textiles. Adv. Mater..

[20] Li X., Zang X., Li Z., Li X., Li P., Sun P., Lee X., Zhang R., Huang Z., Wang K. (2013). Large-area flexible core-shell graphene/porous carbon woven fabric films for fiber supercapacitor electrodes. Adv. Funct. Mater..

[21] Saravanakumar B., Ko T.H., Kim B.S. (2018). Rational design of binder-free ZnCo_2_O_4_ and Fe_2_O_3_ decorated porous 3D Ni as high-performance electrodes for asymmetric supercapacitor. Ceram. Int..

[22] Yuan D., Li B., Cheng J., Guan Q., Wang Z., Ni W., Li C., Liu H., Wang B. (2016). Twisted yarns for fiber-shaped supercapacitors based on wetspun PEDOT:PSS fibers from aqueous coagulation. J. Mater. Chem. A.

[23] Cheng X., Zhang J., Ren J., Liu N., Chen P., Zhang Y., Deng J., Wang Y., Peng H. (2016). Design of a hierarchical ternary hybrid for a fiber-shaped asymmetric supercapacitor with high volumetric energy density. J. Phys. Chem. C.

[24] Ramadoss A., Kang K.N., Ahn H.J., Kim S.I., Ryu S.T., Jang J.H. (2016). Realization of high performance flexible wire supercapacitors based on 3-dimensional NiCo_2_O_4_/Ni fibers. J. Mater. Chem. A.

[25] Tao J., Liu N., Ma W., Ding L., Li L., Su J., Gao Y. (2013). Solid-state high performance flexible supercapacitors based on polypyrrole-MnO_2_-carbon fiber hybrid structure. Sci. Rep..

[26] Ko T.H., Radhakrishnan S., Seo M.K., Khil M.S., Kim H.Y., Kim B.S. (2017). A green and scalable dry synthesis of NiCo_2_O_4_/graphene nanohybrids for high-performance supercapacitor and enzymeless glucose biosensor applications. J. Alloys Compd..

[27] Dong X., Jin H., Wang R., Zhang J., Feng X., Yan C., Chen S., Wang S., Wang J., Lu J. (2018). High Volumetric Capacitance, Ultralong Life Supercapacitors Enabled by Waxberry-Derived Hierarchical Porous Carbon Materials. Adv. Energy Mater..

[28] Saravanakumar B., Jayseelan S.S., Seo M.K., Kim H.Y., Kim B.S. (2017). NiCo_2_S_4_ nanosheet-decorated 3D, porous Ni film@Ni wire electrode materials for all solid-state asymmetric supercapacitor applications. Nanoscale.

[29] Xu Z., Sun S., Cui W., Lv J., Geng Y., Li H., Deng J. (2018). Interconnected network of ultrafine MnO_2_ nanowires on carbon cloth with weed-like morphology for high-performance supercapacitor electrodes. Electrochim. Acta.

[30] Huang J., Chen L., Dong H., Zeng Y., Hu H., Zheng M., Liu Y., Xiao Y., Liang Y. (2017). Hierarchical porous carbon with network morphology derived from natural leaf for superior aqueous symmetrical supercapacitors. Electrochim. Acta.

[31] Pan C., Gu H., Dong L. (2016). Synthesis and electrochemcial performance of polyaniline@MnO_2_/graphene ternary composites for electrochemical supercapacitors. J. Power Sources.

[32] Zhao Z., Shen T., Liu Z., Zhong Q., Qin Y. (2020). Facile fabrication of binder-free reduced graphene oxide/MnO_2_/Ni foam hybrid electrode for high-performance supercapacitors. J. Alloys Comp..

[33] Wu M.S., Chen F.Y., Lai Y.H., Sie Y.J. (2017). Electrocatalytic oxidation of urea in alkaline solution using nickel/nickel oxide nanoparticles derived from nickel-organic framework. Electrochim. Acta.

[34] Peng H.H., Chen J., Jiang D.Y., Li M., Feng L., Losic D., Dong F., Zhang Y.X. (2016). Synergistic effect of manganese dioxide and diatomite for fast decolorization and high removal capacity of methyl orange. J. Colloid Interf. Sci..

[35] Xiao A., Zhou S., Zuo C., Zhuan Y., Ding X. (2014). Controllable synthesis of mesoporous Co_3_O_4_ nanoflake array and its application for supercapacitor. Mater. Res. Bull..

[36] Sun X., Xu T., Bai J., Li C. (2019). MnO_2_ Nanosheets Grown on Multichannel Carbon Nanofibers Containing Amorphous Cobalt Oxide as a Flexible Electrode for Supercapacitors. ACS Appl. Energy Mater..

[37] Zhao J., Li Z., Yuan X., Yang Z., Zhang M., Meng A., Li Q. (2018). A High-Energy Density Asymmetric Supercapacitor Based on Fe_2_O_3_ Nanoneedle Arrays and NiCo_2_O_4_/Ni(OH)_2_ Hybrid Nanosheet Arrays Grown on SiC Nanowire Networks as Free-Standing Advanced Electrodes. Adv. Energy Mater..

[38] Wang X., Liu Y., Arandiyan H., Yang H., Bai L., Mujtaba J., Wang Q., Liu S., Sun H. (2016). Uniform Fe_3_O_4_ microflowers hierarchical structures assembled with porous nanoplates as superior anode materials for lithium-ion batteries. Appl. Surf. Sci..

[39] Cho Y.H., Ko T.H., Choi W.K., Kuk Y.S., Seo M.K., Kim B.S. (2020). Micro-flower NiCoMnO_2_ superstructures prepared by a catalytic chemical oxidation for supercapacitor applications. Tex. Sci. Eng..

[40] Oyedotun K.O., Masikhwa T.M., Mirghni A.A., Mutuma B.K., Manyala N. (2020). Electrochemical properties of asymmetric supercapacitor based on optimized carbon-based nickel-cobalt-manganese ternary hydroxide and sulphur-doped carbonized iron-polyaniline electrodes. Electrochim. Acta.

[41] Du Y., Li G., Chen M., Yang X., Ye L., Liu X., Zhao L. (2019). Hollow nickel-cobalt-manganese hydroxide polyhedra via MOF templates for high-performance quasi-solid-state supercapacitor. Chem. Eng. J..

[42] Bai X., Cao D., Zhang H. (2020). Simultaneously morphology and phase controlled synthesis of cobalt manganese hydroxides/reduced graphene oxide for high performance supercapacitor electrodes. Ceram. Int..

[43] Guo Y., Zhang S., Wang J., Liu Z., Liu Y. (2020). Facile preparation of high-performance cobalt-manganese layered double hydroxide/polypyrrole composite for battery-type asymmetric supercapacitors. J. Alloys Comp..

[44] Nath A.R., Jayachandran A., Sandhyarani N. (2019). Nanosheets of nickel, cobalt and manganese triple hydroxides/oxyhydroxides as efficient electrode materials for asymmetrical supercapacitors. Dalton Trans..

[45] Li M., Cheng J.P., Wang J., Liu F., Zhang X.B. (2016). The growth of nickel-manganese and cobalt-manganes layered double hydroxides on reduced graphene oxide for supercapacitor. Electrochim. Acta.

[46] Wenping Y., Xinyue S., Yan L., Pang H. (2019). Manganese-doped cobalt zeolitic imidazolate framework with highly enhanced performance for supercapacitor. J. Energy Storage.

[47] Kim N.K., Kim W.S., Jang H.J., Chung Y.S. (2020). Fabrication and properties of polyimide-based graphite fibers. Tex. Sci. Eng..

