# Supplementary material for: CoMnO2-Decorated Polyimide-Based Carbon Fiber Electrodes for Wire-Type Asymmetric Supercapacitor Applications
Source: Molecules. 2020 Dec 11;25(24):5863. doi: 10.3390/molecules25245863 (PMC7763561; doi:10.3390/molecules25245863)
Supplement: Supplementary file 1 [file molecules-25-05863-s001.pdf]

Supplementary Materials

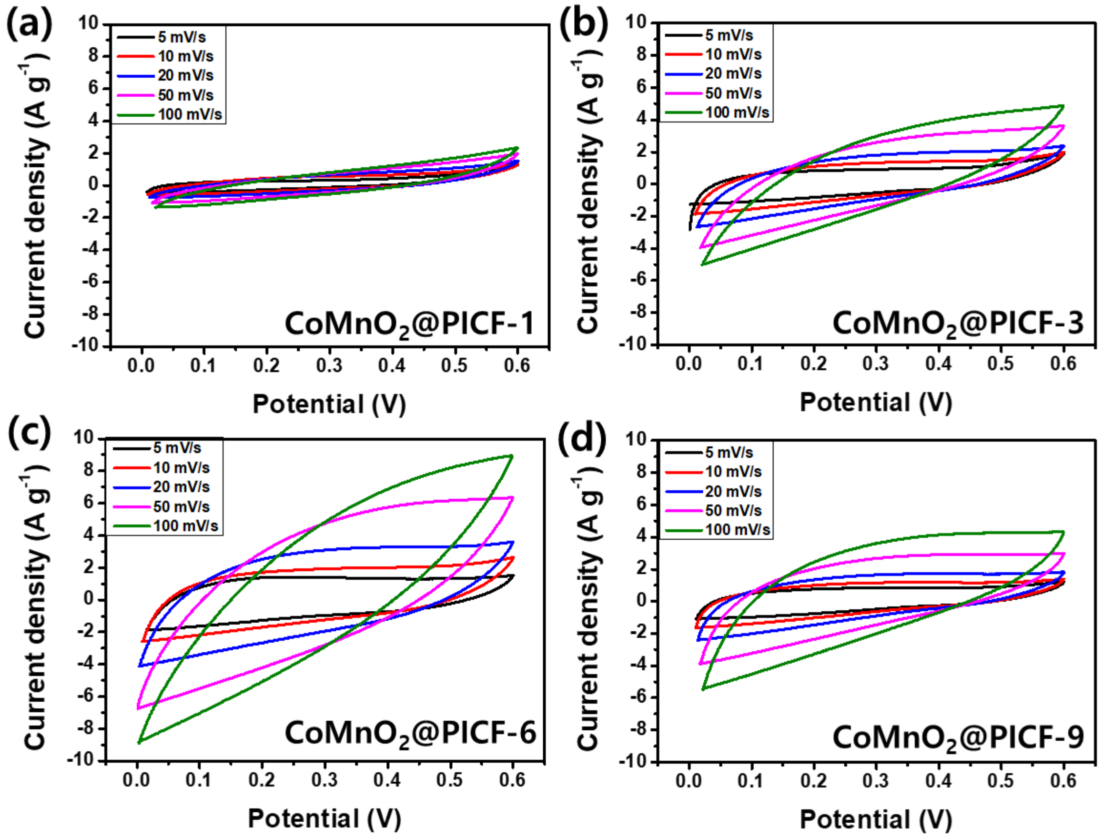

Figure S1. CV curves of $CoMnO_2$@PICF-1 (**a**), $CoMnO_2$@PICF-3 (**b**), $CoMnO_2$@PICF-6 (**c**), and $CoMnO_2$@PICF-9 (**d**) electrode materials at various scan rates.

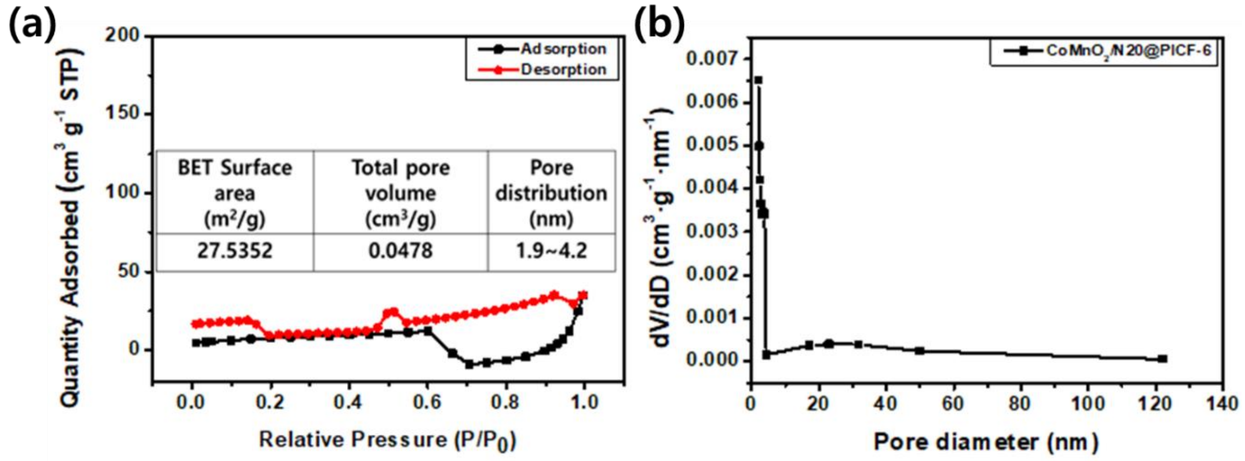

Figure S2. N_2 adsorption/desorption curve (**a**) and the pore size distribution curve (**b**) of $CoMnO_2$/N20@PICF-6 electrode.

Table S1. The specific capacitance, energy density, cycle stability, and electrolyte of the $CoMnO_2$/N20@PICF-6//Fe_2O_3/N20@PICF device, compared to previously reported transition metal oxides based electrode materials.

Electrode Materials	Specific Capacitance	Energy Density	Cycle Stability	Electrolyte	Ref.
NiCoMn-TH/AEG//CFP-S	66 F g^{-1} at 0.5 A g^{-1}	23.5 Wh kg^{-1} at 427 W kg^{-1}	87.8% 10,000 cycles at 6 A g^{-1}	1 M KOH	[40]
NiCoMn-OH//AC	121.5 F g^{-1} at 1 A g^{-1}	43.2 Wh kg^{-1} at 790W kg^{-1}	100% 10,000 cycles at 5 A g^{-1}	PVA/KOH	[41]
CoMn-HW/RGO10//AC	107.6 F g^{-1} at 1 A g^{-1}	38.3 Wh kg^{-1} at 8000 W kg^{-1}	89.5% 3000 cycles at 2 A g^{-1}	3M KOH	[42]
CoMn LDH/PPy//MLG	38.6 mAh g^{-1} at 0.5 A g^{-1}	29.6 Wh kg^{-1} at 500 W kg^{-1}	99.5% 8000 cycles	2M KOH	[43]
NCM//AC	114.5 mAh g^{-1} at 3 A g^{-1}	23.7 Wh kg^{-1} at 2625 W kg^{-1}	93.2% 10,000 cycles	3M KOH	[44]
Ni-Mn LDH/rGO//AC	86.26 F g^{-1} at 1 A g^{-1}	33.8 Wh kg^{-1} at 850 W kg^{-1}	74.1% 10,000 cycles at 10 A g^{-1}	2M KOH	[45]
Co/Mn-ZIF//AC	73.54 F g^{-1} at 0.5 A g^{-1}	52.5 Wh kg^{-1} at 1080 W kg^{-1}	51% 1500 cycles at 10 A g^{-1}	3M KOH	[46]
$CoMnO_2$/N20@PICF-6//Fe2O_3/N20@PICF	221 F g^{-1} at 0.7 A g^{-1}	60.2 Wh kg^{-1} at 490 W kg^{-1}	95% 3000 cycles at 1.4 A g^{-1}	PVA/KOH	This work
